# When and How to Provide Feedback and Instructions to Athletes?—How Sport Psychology and Pedagogy Insights Can Improve Coaching Interventions to Enhance Self-Regulation in Training

**DOI:** 10.3389/fpsyg.2020.01444

**Published:** 2020-07-14

**Authors:** Fabian W. Otte, Keith Davids, Sarah-Kate Millar, Stefanie Klatt

**Affiliations:** ^1^Department of Cognitive and Team/Racket Sport Research, Institute of Exercise Training and Sport Informatics, German Sport University Cologne, Cologne, Germany; ^2^Sport and Physical Activity Research Centre, Sheffield Hallam University, Sheffield, United Kingdom; ^3^Department of Coaching, Health and Physical Education, Faculty of Health and Environmental Sciences, Auckland University of Technology, Auckland, New Zealand; ^4^Institute of Sports Science, University of Rostock, Rostock, Germany

**Keywords:** specialist role coaching, augmented information, constraints-led approach, ecological dynamics, skill acquisition

## Abstract

In specialist sports coaching, the type and manner of augmented information that the coach chooses to use in communicating and training with individual athletes can have a significant impact on skill development and performance. Informed by insights from psychology, pedagogy, and sport science, this position paper presents a practitioner-based approach in response to the overarching question: When, why, and how could coaches provide information to athletes during coaching interventions? In an ecological dynamics rationale, practice is seen as a search for functional performance solutions, and augmented feedback is outlined as instructional constraints to guide athletes’ self-regulation of action in practice. Using the exemplar of team sports, we present a Skill Training Communication Model for practical application in the context of the role of a specialist coach, using a constraints-led approach (CLA). Further based on principles of a non-linear pedagogy and using the recently introduced Periodization of Skill Training (PoST) framework, the proposed model aims to support practitioners’ understanding of the pedagogical constraints of feedback and instruction during practice. In detail, the PoST framework’s three skill development and training stages work to (1) directly impact constraint manipulations in practice designs and (2) indirectly affect coaches’ choices of external (coach-induced) information. In turn, these guide practitioners on how and when to apply different verbal instruction methodologies and aim to support the design of effective skill learning environments. Finally, several practical guidelines in regard to sports coaches’ feedback and instruction processes are proposed.

## Introduction

Coaches endeavor to engage in behaviors that effectively facilitate each athlete’s progress toward achieving particular goals in competition or practice environments. Essential to this progress is athlete learning, and a key tool for coaches is the effective use of verbal instructions and feedback (More and Franks, 1996). Contemporary research has identified verbal instructions are the dominant activity engaged in by coaches at all levels (Potrac et al., 2000; Hodges and Franks, 2002). Different verbal instruction properties, including timing, nature, and intent, have been studied, finding that verbal instruction has important effects on athletes’ learning and performance (Davids et al., 2008; Cassidy et al., 2009; Klatt and Noël, 2019). This considered, the provision of constructive augmented information (including verbal instructions, feedback, praise, and criticism) has long been regarded essential psychological and pedagogical competencies of sports coaches designing learning environments (Holding, 1965; for more recent position statements, see Chow, 2013; Button et al., 2020). Particularly, the rationale for type and manner of verbal communication that coaches choose to use (or not use) with individual athletes can support their skill development and discovery of task solutions and can arguably make a difference for each athlete’s development and successful performance in sports (Partington et al., 2014; Correia et al., 2019). For the purpose of this article, adopting an ecological rationale, augmented information is considered as an instructional constraint on motor learning (Chow et al., 2016; for original insights, see Newell, 1986). This constraint takes the form of verbal feedback and instructions and is delivered by external agents (such as coaches, trainers, sport scientists, teachers, parents, educators, and peers; Handford et al., 1997). With respect to learning experiences, the main goal of verbal feedback and instructions (often in integration with other sensory modalities, such as vision or proprioception) has been stated as follows: “to help educate the attention of a learner to perceive and utilize relevant information sources” within skill (acquisition and refinement) training environments (Correia et al., 2019, p. 126). In support of this goal during learning, it is paramount that sport coaches and teachers have a viable model of practice design that supports the delivery of verbal feedback and instructions to athletes in coaching interventions (see Newell and Ranganathan, 2010; Chow, 2013, for discussions in a non-linear pedagogy and within a constraints-based framework).

From a non-linear pedagogy perspective, because of augmented verbal information being considered as an instructional constraint, pedagogical expertise in deciding *when*, *how*, and *why* to provide *what* verbal information to athletes is crucial. Thus, coaching behavior needs to be based on a comprehensive theoretical rationale for successful implementation and used as part of the learning design in sports coaching. In this article, we introduce a novel Skill Training Communication Model for use of augmented information as an instructional constraint to guide athlete activities during skill acquisition and in preparation for performance in sport. Here, we focus on the use of verbal feedback and instructions in somewhat unique coaching contexts, such as “specialist coaching” (i.e., coaches in charge of one-on-one or small-group trainings to refine athletes’ position-specific skills; Otte et al., 2019a, 2020a).

In order to introduce and underpin the Skill Training Communication Model, this article is structured in three parts. Whereas Parts A and B provide a theoretical foundation for the model in regard of an ecological dynamics rationale for providing augmented verbal feedback during practice (i.e., Part A) and a skill training periodization framework (i.e., Part B), Part C presents the communication model. In particular, this communication model to coaching is further motivated by concerns that traditional coaching strategies and processes often appear to “adhere to established or intuitive instructional methods” (Wulf, 2013, p. 97). Reasons for such concerns include a possible lack of a theoretical framework for providing verbal instructions and feedback in practitioner education programs; this limitation is underlined by the suggestion that there have been “relatively few investigations of coaching” (Partington et al., 2014, p. 404) and that a “body of pedagogically focused coaching research” has only recently begun to emerge (Vinson et al., 2016, p. 54; see also Uehara et al., 2016). Consequently, it is the aim of this article to support coaches in rethinking the role and application of verbal feedback and instructions in a skill training context; this, based on an ecological dynamics rationale to augmented feedback, will be presented in Part A and later be elaborated in Part B [Periodization of Skill Training (PoST) framework] and Part C (Skill Training Communication Model).

## Part A: An Ecological Dynamics Rationale to Augmented Feedback

Feedback and instructions (whether including sources of verbal information, feedback, and/or other modalities) are considered instructional constraints, form augmented feedback (Annett, 1969; Sigrist et al., 2013), and are commonly provided to a learner from external agents during practice and training (Handford et al., 1997). Instructional constraints such as augmented feedback during learning can be distinguished from intrinsic feedback processes that are ubiquitous and naturally occur within individuals engaged in discovery and externally guided learning experiences in representative training environments (Vereijken and Whiting, 1990). While experience of intrinsic feedback (as sensory afferences) during learning is vital, research has shown that externally provided feedback and instructions, or instructional constraints, carefully applied by coaches, may support, guide, and complement learning (Holding, 1965; Newell et al., 1985; Sigrist et al., 2013).

From an ecological dynamics rationale, information regulates action, and practice has been conceived as a search for functional task solutions and relevant performance behaviors, which can become stabilized with experience and learning (Newell, 1991; Handford et al., 1997). Search activities during practice can support the self-regulation of athletes finding high-quality information sources to coordinate their actions. Functional action solutions exist in a landscape of affordances (opportunities for action; Rietveld and Kiverstein, 2014; Strafford et al., 2020), which surround learners in a performance environment (Button et al., 2020). An important role of sport coaches and teachers is to guide the learner’s search of the affordance landscape, and application of instructional constraints is a powerful tool to be carefully used in important search activities (Newell, 1986). Hereby, pedagogical practice is conceived as driving search processes that may be described as “learning to attend to informational variables of the task and modifying actions in terms of informational variables” (Pacheco et al., 2019, p. 3).

The theoretical rationale for using augmented verbal information and feedback to support search activities and guide learners toward functional affordances in the landscape differs considerably from traditional pedagogical models (Davids et al., 2008; Ford et al., 2010). Traditional pedagogies tend to emphasize specific detailed prescription of a movement template for repetitive rehearsal (providing an “optimal” way to perform a specific movement), as well as the application of corrective feedback in repeating a movement technique (Davids et al., 2008). These prescriptive coaching approaches arguably lead to overuse of verbal information and feedback that can impede athlete development by impinging on opportunities for self-regulation (Davids et al., 2008; Partington and Cushion, 2011), which is a major aim of sports training and practice (Handford et al., 1997; Davids, 2015). Therefore, from an ecological dynamics rationale, the careful application or omission of augmented information (i.e., verbally and in integration with other feedback and instruction modes) needs to consider athletes’ self-regulated exploration and search activities.

In the current article, our specific focus is the introduction of a novel Skill Training Communication Model (i.e., in Part C). Particularly, the model aims to support provision of instructional constraints in the context of specialist coaching in team sports (e.g., coaching single athletes and subgroups, such as attackers, defenders, goalkeepers), allowing coaches to individually support and communicate with athletes with specific performance needs. Notably, strategic team tactics (e.g., the coach introducing a tactical game plan to the entire team) that traditionally are adopted in performance preparation in team sports, such as soccer, basketball, volleyball, or rugby, are not the focus of the model. Rather, it is the aforementioned specialist coaching context in team sports that places particular emphasis on the objectives of skill acquisition and refinement in practice designs.

## Part B: The Post Framework

The proposed Skill Training Communication Model (see Part C) builds upon a recently introduced PoST framework by Otte et al. (2019b). The PoST framework, at its core, is focused on how skills are taught by specialist or individual development coaches working with single athletes and/or subgroups of athletes and is based on the theoretical perspective of the constraints-led approach (CLA; Newell, 1986). The CLA considers emerging task, environment, and individual constraints that can change or be manipulated to lead learners to exploit inherent tendencies to “self-organize in attempts to generate effective movement solutions” (Renshaw and Chow, 2019, p. 104; see also Renshaw et al., 2019, for an overview of CLA, allied to principles of ecological dynamics and non-linear pedagogy). In more detail, the specific context of specialist coaching allows practitioners to design training sessions that support a focus on self-organized movement solutions that emerge in the actions of individual athletes with specialized roles in sports teams. In ecological dynamics, it has been proposed that directions of constraints on self-organizing tendencies of individual athletes and sports teams, during synergy formation, are continuously shaped by local-to-global (exploiting intrinsic dispositions for self-organization) and global-to-local influences (being organized by external agents such as coaches; Ribeiro et al., 2019). Particular emphasis in CLA has been placed on exploiting existing local-to-global self-organization processes, which ultimately aim to develop intelligent, self-regulating, and adaptable performers (see Ribeiro et al., 2019; Guignard et al., 2020, for detailed elaborations of bi-directional self-organization processes in team and individual sports). In order to drive these self-regulatory tendencies, it is a major task of sport practitioners to manipulate task constraints within training session designs to facilitate skill learning (Newell, 1985; Pacheco et al., 2019). For example, by adjusting task constraints, such as field sizes, line markings, or practice game rules, coaches can effectively impact athletes’ problem-solving behaviors in finding functional performance solutions themselves; these self-regulating tendencies can emerge, without having to prescribe movement solutions in precise detail for learners. In the constraints-based approach, the coach is not the main problem-solver during practice.

**FIGURE 1:**
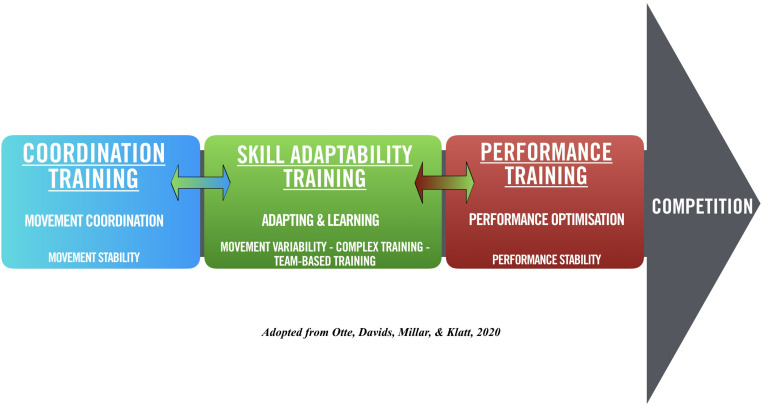
Skill development and training stages according to the Periodization of Skill Training framework.

In terms of skill training planning, the PoST framework displays three broad skill development and training stages that are adapted from Newell’s (1985) Model of Motor Learning; these stages, as presented below in Figure 1, are labeled as Coordination Training, Skill Adaptability Training, and Performance Training (see Otte et al., 2019b, for the detailed theoretical introduction of the framework). The principles of each skill training stage in the PoST framework are a strong guide for the proposed Skill Training Communication Model for specialist coaches to be able to carefully apply various forms of feedback and instruction at each skill training stage. Verbal communication induced by coaches may predominantly be seen as augmented information acting as an instructional constraint to guide learners’ search and problem-solving activities (Davids et al., 2008); this is in order to stabilize functional coupling of perception and actions within the specific training environment: the foundation of skilled performance (Newell, 1991; Newell and Ranganathan, 2010; Correia et al., 2019).

Altogether, the CLA presents an emerging and contemporary perspective on skill acquisition and specialist coaching approaches by (implicitly) affecting athletes’ goal-directed behavior through the design of training sessions. Constraint manipulation arguably forms the primary coaching approach toward shaping skill learning during practice, and it is particularly important to consider how complementary, augmented verbal feedback and instructional constraints can be used to guide athletes’ search for functional solutions. In the following part of this article, we aim to provide guidance for practitioners to consider how and when to apply appropriate feedback and instruction forms within a particular skill training context via the Skill Training Communication Model.

## Part C: The Skill Training Communication Model

**FIGURE 2:**
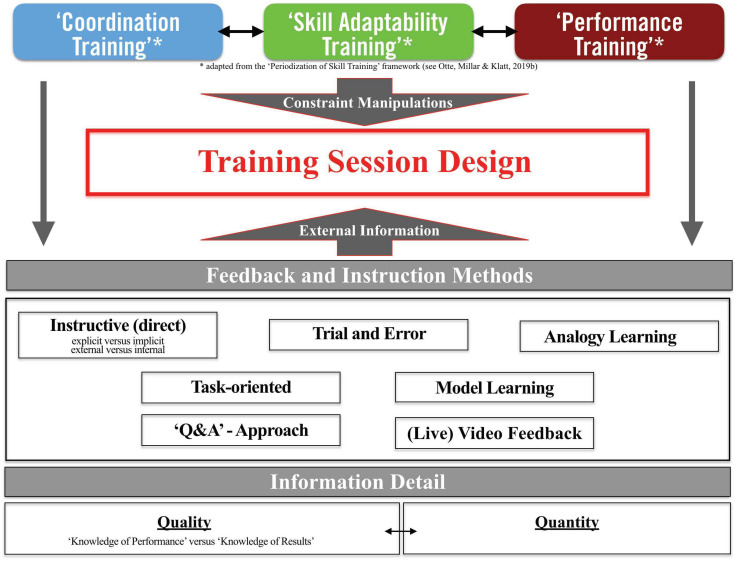
The Skill Training Communication Model in specialist sports coaching.

As an extension of the PoST framework, Figure 2 proposes a novel Skill Training Communication Model that presents a multifaceted structural approach to planning effective training session designs (i.e., a core task for sport coaches and displayed by the red box in the center of Figure 2). In more detail, the proposed structural approach considers (1) the *athlete’s skill training stage* (as displayed by three training stages at the top of the figure); (2) *feedback and instruction methods* [e.g., question-and-answer (Q&A) approach and model learning]; and (3) *information detail* in terms of quality and quantity (i.e., bottom part of Figure 2).

According to the Skill Training Communication Model, in order to plan effective training session designs, coaches should follow a stepwise approach. First, coaches would consider the athlete’s skill training stages that work to directly impact the manipulation of constraints and the overall training session design (e.g., regarding levels of game-representativeness and task complexity in training; Otte et al., 2019b). In simple terms, the training design is the main pedagogical method for skill learning; for example, athletes in the Coordination Training stage (see below) may be confronted with simplified training tasks that (without verbal feedback and instruction) themselves drive exploration of, and search for, functional movement solutions. Second, coaches’ choices of augmented verbal information would be affected by athletes’ skill training stages (i.e., athletes in different skill training stages should experience different methods of verbal communication). In turn, the skill training stage and training session design will be complemented by feedback and instruction methods, providing external information. These methods are embedded into the training session and support critical task constraint manipulations; for example, feedback and instructions provided to athletes in the Coordination Training stage complement the training design in that coaches (verbally) guide aforementioned discovery and search processes.

### Skill Training Stages

The top section of the Skill Training Communication Model shows how each specialist coach needs to start with an understanding of the athlete’s current skill development and training stage for a macrocycle (i.e., multiple training months), a microcycle (i.e., one training week), or a single training session (see Otte et al., 2019b). Starting with the athlete’s current training stage affords individualized training sessions, where the individual is coached according to his/her specific needs. In order to place the athlete within a specific skill training stage and, later, to select the most fitting feedback and instruction methods, the framework differentiates between three distinct stages (i.e., the Coordination Training, Skill Adaptability Training, and Performance Training stages).

#### Coordination Training Stage

Athletes in the Coordination Training stage are at a developmental level, with a primary need to stabilize general coordinative movement patterns during performance within game-representative environments. Here, athletes are encouraged to search and explore movement patterns by (during playful activities and games) learning to exploit intrinsic self-organizing motor system degrees of freedom (e.g., body segments, muscles, and joints; Uehara et al., 2016; Correia et al., 2019). The primary aim at this stage of learning design is exploratory activity by athletes. Exploratory movements are required to perceive relations between system degrees of freedom (roughly, components of the body) and between information and action. Learning experiences at this stage of development should provide opportunities for learners to perceive novel affordances that can be achieved by particular action patterns. With respect to skill development, this idea was elegantly expressed by Adolph and Justin (2019), who harnessed Harlow’s (1949) notion during motor development that individuals do not really learn to move, rather they are “learning to learn to move.” To encourage exploratory practice in athlete development, the acquisition of functional sport-specific actions, through simplified tasks and coach-supported constraint manipulations, is prominent at this stage (Otte et al., 2019b).

#### Skill Adaptability Training Stage

During Skill Adaptability Training, the focus lies on perceptual-cognitive regulation of adaptive actions in more complex and varied learning environments. In this regard, the PoST framework proposes three skill training substages termed Movement Variability Training, Complex Training, and Team-Based Training (see Otte et al., 2019b, for practical application of these training stages). Training designs with appropriate levels of game-representativeness and task complexity are used in the (re)organization of functional perception-action couplings, comprised of non-linear and dynamic individual, task, and environment constraint interactions (see Hüttermann et al., 2019; Renshaw et al., 2019). Consequently, the advancement of perceptual-cognitive skills to regulate robust and adaptable movement coordination is the primary goal (Ford et al., 2010; Renshaw et al., 2019).

#### Performance Training Stage

Performance Training, as the third developmental stage, is focused on preparing athletes to apply the acquired self-regulatory skills (technical-tactical, physical, and psychological) in competitive performance. The main focus is on the preparation of individual athletes through exposure to representative training designs for high-pressure competition. This greater performance-driven focus may highlight the importance of athletes’ preparation of perception, cognitions, and actions for competition (e.g., including mental readiness, match fitness, and confidence as important factors for athletes’ performance; Ford et al., 2010; Otte et al., 2020b). Notably, this training stage mostly considers competitive environments in professional sports organizations (e.g., performance preparation immediately preceding a major competitive soccer game). While developing athletes (as part of their skill learning and development) need to be exposed to these challenging constraints on carefully considered and limited occasions, it is important not to overdo these experiences. Limited exposure is needed in development because of the high intensity of these practice constraints and to avoid detrimental negative experiences on confidence and to manage expectations at this training stage (Otte et al., 2020a). For example, a young performer may be asked to play up a grade or to sit on the bench as a substitute in a competitive senior game. Limited game time (in the order of minutes) may be provided after careful consideration by the coaching support staff.

### Feedback and Instruction Methods

**FIGURE 3:**
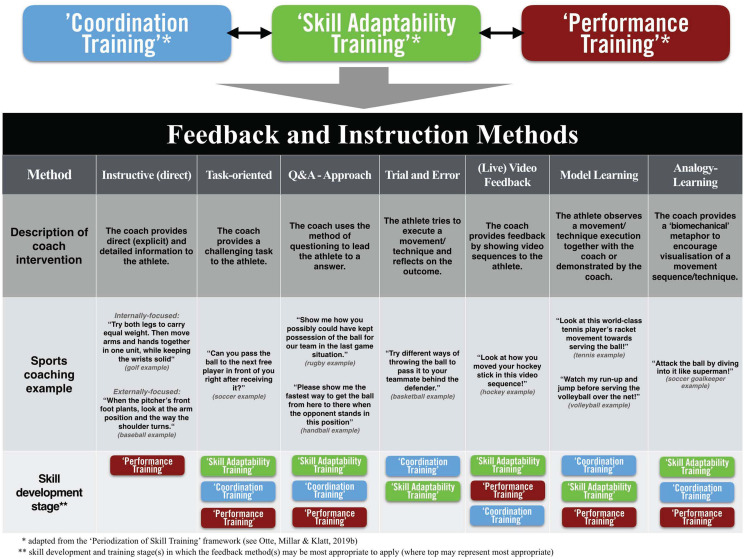
Description table of feedback and instruction forms and methods in relation to the skill training.

As introduced in the Skill Training Communication Model (i.e., see Figure 2), a categorical distinction for verbal feedback and instruction approaches may be made between various methodologies (e.g., task-oriented communication or analogy learning). Depending on the individual athlete’s skill training stage and/or the training activities undertaken, different feedback and instruction methods have to be considered by specialist coaches to support effective skill development. Closer descriptions of these communication methods are elaborated in the following sections and displayed in Figure 3 below. In detail, Figure 3 presents (1) a description of the *coaching intervention for each feedback/instruction method* (i.e., the third row from the bottom), (2) practical *sports coaching examples* for each communication method (i.e., the second row from the bottom), and (3) the *proposed skill training stages*, which could be predominantly considered by coaches for a coaching intervention (i.e., the bottom row in Figure 3). Notably, while major aspects of feedback and instruction are provided verbally, this acoustically based communication approach may direct athletes’ perception toward more visual and haptic modalities (e.g., verbal feedback as part of multisensory analogy learning). In turn, there is the notion that for some skill training contexts an integration of different communication methods is inevitable, and furthermore, it could be an effective strategy for providing optimal practice and learning conditions for athletes (e.g., Klatt and Smeeton, 2020; Klein-Soetebier et al., 2020). Consequently, the following sections will present and elaborate on seven feedback and instruction methods of *instructive (direct) verbal communication*; *task-oriented communication*; *Q&A feedback*; *trial and error*; *(live) video feedback*; *model learning*; and *analogy learning.* Notably, presented feedback forms have been selected based on multiple authors’ experience of sports coaches commonly applying these instructional constraints to practice environments.

#### Instructive (Direct) Verbal Communication

The instructive method, whereby the coach gives direct, prescriptive, and corrective verbal instructions to the athlete, is perhaps considered to be the most widely applied, traditional form of instructional constraint used in coaching (Davids et al., 2008; Uehara et al., 2016; Correia et al., 2019). However, verbal information should instead be mainly used as an augmented informational constraint to guide an athlete’s search activities. When learning to learn to move, it is the athlete who needs to use information to solve a performance problem and not the coach providing verbal information to solve the problem for an athlete. This pedagogical method is synonymous with an athlete-centered approach to coaching. Consequently, outside the Performance Training stage (where immediate performance is supported under time constraints), directing and prescriptive verbal instructions should be reduced to a minimum (Williams and Hodges, 2005; Ford et al., 2010; Button et al., 2020).

There is a significant body of research that often differentiates augmented verbal information (provided by the coach) into (i) explicit and implicit and (ii) internally focused and externally focused information (Poolton and Zachry, 2007; Lam et al., 2009; Sigrist et al., 2013; Wulf, 2013; Winkelman, 2020). Whereas explicit information constitutes verbal communication containing a lot of detailed information, implicit information describes communication that is associated with implicit learning by athletes, in the absence of detailed (technical) information on movements of specific limb segments and joints of the body (Masters, 2000; Jackson and Farrow, 2005). Notably, both explicit and implicit approaches are highly interdependent and often intertwined in the learning process (Hodges and Franks, 2002; Poolton and Zachry, 2007). Regarding internally focused (or body-focused) augmented information, feedback and instructions directly target the athlete’s body parts and specific movements (e.g., coach: “Look at your toes and the angle of 20° at which they should point!”). In contrast, externally focused (or outcome-focused) feedback and instructions focus on effects of movements on the environment (e.g., coach: “Try to flatten the flight curve of the ball in the air and make it spin back after the bounce!”).

What does this body of work imply for coaching practice? First, explicit and detailed verbal instructions may constrain and impede performers in attending to and perceiving relevant information and opportunities for action within the learning environment; these information sources would “support the search for functional performance solutions for their specific task goals” (Correia et al., 2019, p. 126). If the main role of instructional constraints and augmented verbal information is to guide athletes’ search during practice, providing large amounts of explicit verbal feedback and instructions, especially immediately following skill performance, may curtail and hinder intrinsic feedback system function during self-organized exploration for functional movement solutions.

Second, explicit–internal information and the conscious reinvestment in (technical) movement knowledge that could potentially result from it could hinder the athlete’s implicit perceptual–motor regulation during action (see Masters and Maxwell, 2008, for a theoretical overview of the theory). Consciously attending to one’s own movements during self-regulated actions may disturb the functioning of perception–action couplings (Masters, 1992; Poolton and Zachry, 2007; Renshaw et al., 2009). In contrast, athletes who receive more implicit feedback are shown to be demonstrably more effective and efficient in movement regulation (Wulf and Prinz, 2001; Wulf, 2016). Notably, this view has been supported by a large amount of research from multiple sport contexts, such as dribbling tasks in soccer and hockey, putting tasks in golf, batting tasks in baseball, and climbing tasks (e.g., see Masters and Maxwell, 2008).

Third, and in conjunction with the previous points, when under pressure, athletes with detailed declarative movement knowledge rather tend to choke (see Hill et al., 2010, for a review on choking in sport). On the contrary, athletes who had experienced significant amounts of implicit learning were found to be more resistant to perturbations from pressure in their performances (Masters, 2000; Masters and Maxwell, 2008).

Finally, and in order for athletes to use exploratory behaviors in practice and freely self-organize movement solutions, with little consideration of explicit movement details, verbal feedback and instructions should be limited to a minimum in the Coordination Training and Skill Adaptability Training stages. However, in the preparation of athletes for competitive performance, time constraints in the build-up to an event may require more direct and explicit coaching approaches. There is less time for discovery learning and exploratory behaviors at that stage of performance preparation. This is because, in the Performance Training stage, skill learning is not the major objective, but rather prepare athletes to compete in an event or match. At this stage, underpinned by the developmental work already undertaken, coaches may need to communicate verbally in a direct way, implementing a focused, task-oriented coaching method, especially when supporting athletes’ adaptation to changing environmental or tactical constraints of a specific competitive event. Nevertheless, it is still important for coaches to use instructional constraints sparingly and avoid overburdening athletes with needless, verbal instructions that are not needed in athletes’ decision-making during performance. The use of instructional constraints should still support athletes’ self-regulation (i.e., perception, cognition, problem-solving, decision-making, and actions), but in a focused manner related to searching processes within a specific competitive environment or event.

#### Task-Oriented

With focused task-oriented coaching, the coach initially tries to challenge the athlete by providing a task (e.g., a coach setting a movement task for a hockey player: “Can you open your body toward the full field with your first contact when receiving the ball?”). While this task is delivered verbally by the coach, from an athlete-environment-centered perspective, it demands performers to explore action solutions via visual or haptic senses and thus to directly perceive interactions. Further, this task-oriented approach does not aim at specifying *how* an athlete performs an action (Pacheco et al., 2019). Rather, this approach appears to be more focused, task-orientated, and goal-directed in order to assist athletes in finding more functional task solutions (Pacheco et al., 2019). Especially, in the Coordination Training and Skill Adaptability Training stages, if the athlete is unable to accomplish a task after several training attempts, the integration of further implicit and guiding feedback and instruction forms may be an option to guide the athlete’s search activities (Hodges and Franks, 2002; Williams and Hodges, 2005).

#### Q&A Approach

The Q&A approach or questioning (divergent or convergent in nature) appears to be another suitable method of verbal feedback for reflection and self-learning (Schoön, 1987; Williams and Hodges, 2005; Partington et al., 2014; Vinson et al., 2016). Linked to Mosston (1966, 1992) spectrum of teaching styles (e.g., guided discovery), the Q&A approach may take various forms in which the coach may apply sequences of (systematic) questions to drive athletes’ discovery of a (codetermined) target. While there is a need to critically review potential overemphases of teacher-driven decision-making and problem-solving for the learner, a merit of Mosston’s proposed teaching styles (Metzler, 1985; Goldberger et al., 2012) is that reciprocal and divergent discovery styles are aligned with the athlete-centered coaching perspective promoted by an ecological dynamics rationale proposed in this article.

It is also of relevance that, in an ecological dynamics rationale, questioning methodology used by a coach needs to be responded to by an athlete’s actions, not verbal responses. With respect to this crucial differentiation between emergent actions and verbal descriptions in practice, it is important to note that Gibson (1966) distinguished between “knowledge of” and “knowledge about” the environment. On the one hand, in sport, knowledge of the environment supports functional actions (see Arauìjo and Davids, 2011). On the other hand, knowledge about the environment facilitates symbolic representational understanding, which may be exemplified by understanding of shapes and patterns on a tactical white board. The aim of a sport practitioner’s attempt to provide questioning should be targeted at developing knowledge of a performance environment, which may stimulate an athlete’s self-regulatory activities in practice. In turn, the aim of a sport practitioner’s use of questioning should always be to elicit an action, not a verbal response. The coach may try to guide the athlete to the desired answer in an implicit and external way (e.g., a coach guiding a handball player to self-reflect on the past play during practice: “Show me how you could handle the last 1-versus-1 (1v1) situation differently, when you’re pressured by an opponent and trying to find your open teammate in space”). Further, a focus on action-scaled affordances, constrained by athletes’ action capabilities in emerging environments (see Fajen et al., 2008), may affect coaches’ verbal phrasing of questions; for example, a basketball coach asking an athlete to reflect on the possibility of performing an action could say: “How did you time your run toward catching the bounce pass quicker this time, compared to the last pass that went out-of-bounds?”

Predominantly in the Skill Adaptability Training and Coordination Training stages, these latter two approaches of task-oriented coaching and Q&A feedback may be of great value for athlete-environment-centered coaching and the search for and exploration of functional movements and solutions to tactical problems (O’Connor et al., 2017). Particularly, time restrictions in these training stages usually appear to be rather low and the specialist coach (by using “higher order questions,” such as why and how; O’Connor et al., 2017) provides an opportunity to reinforce an interactive and detailed exchange with the athlete(s) to guide further exploratory and discovery activities in practice and performance.

#### Trial and Error

In the perspective of the “trial and error” approach, it is a mixture of verbal, visual, proprioceptive, and haptic information that athletes are facing. While searching for functional solutions by designing training sessions with rich affordance landscapes, players could be further alerted to the presence of key information sources through a limited number of verbal informational constraints (Davids et al., 2008).

First, it is important to note that the training session design aims to be the main stimulus for promoting athletes’ search, exploration, and learning behaviors. Particularly, through constraint manipulations and the credo of “repetition without repetition”, coaches could follow an implicit and tacit approach toward using instruction and feedback (Bernstein, 1967; see Otte et al., 2020a, for training examples); this approach highlights principles of local self-organization of actions and places a dominant focus on training designs supporting expansive search for, and attunement to (performance-representative), contextual information emergent in competitive environments (Horn et al., 2007; Seifert et al., 2019).

Second, verbal information provides valuable assistance in constraining an athlete’s exploratory behaviors, problem-solving, and self-discovery of “the relationships between cues/movement patterns and behavioral outcomes” relatively freely (Jackson and Farrow, 2005, p. 315). For example, a coach encouraging a hockey player to attempt the forehand shot during practice could manipulate task constraints “driving” the shooter toward the forehand side for him/her and providing an instructional constraint by saying, “Just try this shooting movement and see how it feels!”. The goal of this approach remains for players to self-organize and explore their own movements and through their experiences to receive intrinsic feedback on the effectiveness of their movement attempts; this feedback on the task outcome may often be based on perceiving intrinsic information through visual, proprioceptive, and haptic systems. Moreover, because this feedback method highlights the importance of discovery, self-monitoring, and the self-organization of movement patterns, the coach adopts the role of a facilitator. Specifically, a facilitator would avoid using direct explicit verbal feedback and follow a “hands-off” strategy in learning (Handford et al., 1997; Chow, 2013; Light and Harvey, 2015; Uehara et al., 2016; Correia et al., 2019).

Altogether, this feedback method appears to be suitable for skill training in the stages of Coordination Training and Skill Adaptability Training; this is due to a focus on athlete self-organization and movement variability in these (de)stabilized training stages.

#### (Live) Video Feedback

(Live) video feedback, as a technological feedback medium, represents another possible method of feedback that provides an effective (real-time) tool for coaches around a training session or competition (Williams and Hodges, 2005; Davids et al., 2008; Ward, 2011). On the one hand, the visualization of training/game sequences (in the best case recorded from a point-of-view camera shot) can prove helpful in the Coordination Training and Skill Adaptability Training stages. For examples, studies in sports such as gymnastics, swimming, and volleyball found increased skill performance in response to coaching interventions including self-video feedback (e.g., Hazen et al., 1990; Winfrey and Weeks, 1993; Zetou et al., 2002; Boyer et al., 2009). Here, this visual self-feedback may not include additional verbal guidance by coaches. On the other hand, clearly targeted verbal feedback, complemented by video footage of an athlete’s exploration and (movement) solutions, can support specific search activities in the Performance Training stage. For example, professional soccer clubs began using large video walls at their training facilities for immediate playback of patterns of play in practice (Bundesliga, 2018); these oversized video screens particularly underline how a global-to-local direction of synergy formation in sports teams can be supported by augmented verbal and visual information in performance preparation. Additionally, this performance-driven use of video feedback may be delivered in various forms, such as (opposition) team, individual skill, or motivational videos, which may further be accompanied with statistical performance data (e.g., pass completion rates or shot percentages; see O’Donoghue, 2006, for an overview).

#### Model Learning

Model learning or observing holistic movements together with the coach can be considered a building block of visually induced information for guiding athletes’ search activities (Scully and Newell, 1985; Scully and Carnegie, 1998; Correia et al., 2019). Scully and Newell (1985) showed how visual informational constraints from models guided the actions of learners in motor learning. By perceiving and imitating a model’s relative motion pattern (e.g., the relations between body parts), athletes are afforded with constraining augmented information to facilitate their search for functional task solutions (Newell et al., 1985; Scully and Newell, 1985). In other words, model learning may act as a *rate enhancer*, rather than a rate limiter, in early skill acquisition stages, such as the Coordination Training stage with a focus on athletes’ exploration for stable movement coordination (e.g., see Al-Abood et al., 2001a, b). Here, evidence further suggests presenting learners with models of movement patterns of different performers at different performance levels, to showcase a range of movement possibilities in the affordance landscape (Al-Abood et al., 2002). Specifically, strategies regarding (expert) video modeling before and after skill performance have been considered by previous research; for example, studies on video modeling in sports, such as tennis, wall climbing, basketball, and volleyball, showed enhanced movement performance following this video intervention (e.g., Scott et al., 1998; Harle and Vickers, 2001; Boschker and Bakker, 2002; Zetou et al., 2002). Further, active, on-field demonstrations and “freezing strategies” (i.e., freezing skill training exercises or play) by coaches could additionally constrain the perceptual search space and help attune athletes to visual information for functional movement solutions (Pacheco et al., 2019).

Overall, model learning (including demonstrations) appears to be apt for learning and the search of specific movement solutions. In other words, these forms of visual instructional constraints during coaching interventions appear to be particularly effective for athletes acquiring sport-specific and novel movement patterns (i.e., in the Coordination Training stage; Al-Abood et al., 2001a, b) and athletes seeking to attune to relevant information variables (i.e., in Skill Adaptability Stage). Notably, and based on a single athlete’s intrinsic dynamics, coaches should highlight the existence of a multitude of reliable and dynamically stable movement patterns and solutions for a task (Newell and Ranganathan, 2010); this approach stands in contrast to traditionally advocated idealized technical movement solutions promoted by coaches (e.g., see Otte et al., 2019a, for findings in the specialist soccer goalkeeper coaching context).

#### Analogy Learning

In addition to the former communication method of model learning, movement analogies (also termed as “biomechanical metaphors”; i.e., a verbal illustration and visualization of a movement) can provide a valuable feedback alternative for coaches (e.g., Hill et al., 2010; Newell and Ranganathan, 2010; Fasold et al., 2020). For example, the statement “your arms and hands could build a wall from which the ball bounces back into the other team’s court” could be one movement analogy for a “blocking” action in volleyball.

Despite analogies representing verbal forms of communication, these augmented informational constraints potentially direct the search activities of an athlete toward an external focus of attention, a previously experienced feeling (e.g., “imagine throwing a frisbee” for a one-handed backhand return in tennis), and contribute an additional, strong visual value; thus, analogies have the potential to be subconscious to the perceiver and/or promote implicit learning, which is more resistant to forgetting or emotional perturbations (Poolton et al., 2006; Poolton and Zachry, 2007; Renshaw et al., 2009; Williams and Ford, 2009; Newell and Ranganathan, 2010). In detail, Winkelman (2020) recently proposed three categories of analogies for providing visual information to support movement performance: (1) scenario-based analogies (i.e., the consideration of an analogous scenario, such as the well-known “reaching for the cookie jar” analogy for a basketball throw); (2) constraint-based analogies (i.e., perturbation or channeling of information on movement performance, such as “you have resistance bands in your knee joints that constantly pull you down slightly” to guide a volleyball player’s set position); and (3) object-based analogies (i.e., featuring an inanimate object onto, e.g., a soccer GK’s movements: “make a scoop net with your arms and hands to intercept a low shot rolling toward you”; “make a wide wall with your arms, legs, and trunk to block any shot that may be low or high”). All of these categories establish fruitful arrays for coaches to transfer explicit verbal information into an arguably more relatable and effective form for athletes in various skill training stages. Consequently, athletes in the training stages of Skill Adaptability Training and Coordination Training may particularly benefit from analogy learning.

### Information Detail

In the last part of the Skill Training Communication Model (see bottom part of Figure 2), the coach selects the degree of information detail to be communicated to athletes. From a more applied coaching perspective, the quality and quantity of information play crucial roles and need to be considered in perspective of the athlete’s development stage.

#### Information Quality

The quality of augmented information could also be related to traditional concepts such as “knowledge of performance” (KP) and “knowledge of results” (KR; see Johnson and Proctor, 2017, for a review on feedback in skill acquisition and training). First, KP provides information on movement performance or processes during the motor skill execution (e.g., kinetic feedback on forces applied during the movement or kinematic feedback on spatial and temporal properties of the movement; Johnson and Proctor, 2017). Notably, this information may not solely be aimed at the past state of the movement dynamic; it may be regarded as *transition information* that focuses on the control of the performance solution that facilitates the transition to a new pattern of coordination (see Newell, 2003). Transition information may target feedback regarding athletes’ changes over different timescales in organization and transitions between various movement patterns; these changes form an integral part of emerging sport contexts and non-linear learning (Chow, 2013; Orth et al., 2018a, b). Second, KR provides rather externally focused information on task outcomes (e.g., information provided to athletes on whether the task goal was achieved or the degree of error that led to lack of achievement; Williams and Hodges, 2005; Winkelman, 2020). Based on the athlete’s focus to search for functional task and movement solutions, coaches have opportunities to provide informational constraints to athletes through both KP (e.g., through movement-related analogies) and KR (e.g., through the training session design and constraint manipulations). The latter information on KR may be further underlined by extrinsic feedback through external sources that stands in contrast to intrinsic feedback (i.e., the athlete’s own attunement to perceptual information emerging from movement performance). Specifically, through (objective) performance analytics data compiled from motion tracking devices or sensors, coaches in high-performance sports increasingly have the opportunity to include extrinsic feedback sources into their coaching. For example, high-quality GPS data on individual players’ sprinting speeds and running distances within soccer games may be used by coaches to globally guide synergy formation between teammates. However, a challenge is to avoid athletes becoming overdependent on augmented information rather than becoming highly attuned to information from intrinsic feedback systems to solve movement problems (Handford et al., 1997).

In order to provide more distinction to the quality of information provided by coaches, the concepts of KP and KR may be further embedded into goal-directed *categorical* (i.e., correct/false), *graded* (i.e., the degree of correctness of a movement solution), and *detailed information* (i.e., degree of correctness along with detail information) (e.g., Luft, 2014; Johnson and Proctor, 2017). In the Coordination Training and the Skill Adaptability Training stages, it may often make sense to (if at all) solely provide brief categorical or graded feedback (e.g., “too slow,” “too fast,” “too high,” “too much spin”). Particularly, aforementioned action-scaled affordances may support key coaching points in these training stages. By directing feedback toward external information (e.g., the sprinting distance and speed needed to receive an air pass in American Football), simple cues provided to athletes could aim to guide athletes’ search processes. Moreover, this reduced communication approach should be delayed in order to allow an athlete to provide his/her own performance estimate before directing the athlete’s attentional focus toward discovery and self-organization of functional movement patterns and task solutions (Hodges and Franks, 2002; Davids et al., 2008; Sigrist et al., 2013). However, in the Performance Training stage and potentially in later parts of the Skill Adaptability Training stage (e.g., when working with more advanced performers), graded feedback or detailed (extrinsic) feedback on the performance and/or the results may be deemed as more appropriate. For example, a coach providing feedback to a soccer goalkeeper defending the goal could say: “Watch the distance between the approaching attacker and yourself—once the attacker dribbles inside the box, defending a close distance 1v1 situation will be your task” (for evidence of goalkeeper’s use of time to contact information with an attacker in 1v1 dyads, see Shafizadeh et al., 2016). Note that there is no specification of precisely how an athlete should solve a performance problem using that exemplar feedback, because the wording is used to stimulate further exploration of a specific affordance (which can be for “good or ill” as noted by Gibson, 1979).

Finally, information quality may be judged in terms of different levels of *emotional value*. For example, feedback for athletes could be positive and supportive, in that it is praising, motivating, and constructive, or rather negative, in that it is critical or scolding (Smith and Cushion, 2006; Ford et al., 2010; Luft, 2014). Notably, supportive feedback and praise for performance outcomes, improvements, and efforts should be prioritized, whereas negative feedback should be limited (Smith and Cushion, 2006; Sigrist et al., 2013).

#### Information Quantity

Information quantity may be constituted of two components: the *timing of feedback* (e.g., before, during/concurrent or after the action/training/game) and the *feedback frequency* (e.g., during/after each attempt, in regular intervals or randomly) (e.g., Hodges and Franks, 2002; Luft, 2014). First, in terms of *feedback timing* before and during skill execution, practitioners may be cautious of not providing large amounts of movement-related, verbal information to athletes in the Skill Adaptability Training and Coordination Training stages. Because a key aim of practice designs is to facilitate athletes’ self-regulation tendencies, provision of verbal feedback should also not occur *immediately after* an action sequence in training. Provision of too much verbal feedback, especially immediately after a movement response, is a form of “overcoaching,” as previously stated, and has been shown to negatively restrict movement exploration (Davids et al., 2008), possibly inhibiting players’ own decision-making abilities (Smith and Cushion, 2006; Ford et al., 2010). In further support of this notion of delaying verbal feedback, studies have shown expert athletes to judge own performances more accurately than their coaches (e.g., see Millar et al., 2017, for an investigation into athlete–coach agreement on boat speed in rowing). Hence, athletes’ intrinsic and self-directed feedback for own performances may provide enough information to drive skill learning in certain skill training stages.

Second, low *feedback frequency* for athletes in the Coordination Training and Skill Adaptability Training stages may be sufficient, mainly due to previously highlighted search and discovery processes for functional movement solutions. Additionally, coaches giving less frequent feedback would be able to spend longer time periods on (silently) observing the athletes, which may be helpful in order for practitioners to monitor athletes’ (functional) perception–action couplings and individual capacities and assess the overall quality of the designed training environment (Smith and Cushion, 2006; Correia et al., 2019). Notably, in the Performance Training stage, feedback may be required more frequently than at developmental stages of learning; this, and only if athletes need the verbal intervention information, is due to time constraints and the apparent performance focus under immediate competitive pressure. Here, it still may be argued whether this feedback would need to be given as part of an explicit and internally focused verbal coaching intervention. In order to assist athletes’ search and exploration for functional movement solutions, simple prompts, cues, and questions may display verbal alternatives for guiding athletes’ search activities (O’Connor, 2012).

## Concluding Remarks

Overall, this article pursues the goal of presenting a conceptual Skill Training Communication Model. In order to follow the call for “more practitioner-based articles in coaching journals […] showing how goal setting and performance feedback procedures can be adopted” (Ward, 2011, p. 109), this position article aims at integrating academic knowledge with practically applicable feedback and instruction forms for various specialist coaching contexts (i.e., coaches focusing on sport-specific skill acquisition and refinement when working individually with single athletes or subgroups of athletes). The presented theoretical and practical insights underline the need for specialist coaches to display great levels of psychological and pedagogical expertise on how and when to (purposely not) provide external feedback and instructions to individual athletes in training and competition environments.

Finally, the Skill Training Communication Model hopes to inspire future research in the field of sports coaching. Additionally, the article aims at supporting sports coaches by providing the following feedback and instruction guidelines:

1. The training design that facilitates athletes’ self-regulation in sport performance should always be at the core of all learning and coaching activities. By developing representative training sessions and manipulating relevant task constraints, coaches can most effectively drive athletes’ search processes that, in turn, provide highly valuable intrinsic feedback for athletes; this type of feedback is essential for supporting self-organization tendencies for functional movement solutions in response to game-related problems.
2. The coach’s understanding of the athlete’s particular skill development and training stage is paramount for appropriate selection of feedback and instruction methods. Especially the stages of Coordination Training and Skill Adaptability Training may (if at all) demand more implicit haptic and visual feedback forms (e.g., including methods, such as analogy learning, model learning, and video feedback). This stands in contrast to the Performance Training stage, which due to immediate performance and time pressure may require coaches to apply a more targeted and direct communication style.
3. An increased amount of feedback and instructions (in terms of information quality and quantity) likely is not more beneficial for athletes. In contrast to the common notion, “the more, the better”, athletes at particular skill developmental stages actually benefit more from self-regulatory approaches and minimized explicit feedback and instructions used sparingly (Jackson and Farrow, 2005).
4. Related to Point 3, the timing of visual feedback is also important in order for athletes to perceive and use intrinsic information from movements to self-regulate in solving ongoing performance problems. Coaches should delay the provision of augmented feedback in order to provide time for athletes to perceive movement feedback for use in ensuing practice tasks (Button et al., 2020).
5. Augmented verbal information should avoid a specification of precisely how an athlete should solve a performance problem. The wording of feedback and instructions is used to stimulate and elicit further exploration of specific opportunities for action. Consequently, the coach is not the main problem-solver during practice (i.e., by directly verbalizing the performance solution to the athlete) and rather acts as a “moderator” to guide athletes’ search and problem-solving for functional (movement) solutions.
6. The feedback and instruction methods that athletes seek and the way that individual athletes respond to these should drive coaches’ communication. In this respect, an “understanding of athlete-centered coaching is necessary” (Côté et al., 2010, p. 64), and thus individualized feedback and instruction approaches should also consider each individual athlete’s preferences.

## Data Availability Statement

The original contributions presented in the study are included in the article/supplementary material. Further inquiries can be directed to the corresponding author.

## Author Contributions

FO developed the conception of the model and wrote the first draft of the manuscript. KD, S-KM, and SK contributed to re-design and presentation of the model and wrote sections of the manuscript. All authors contributed to manuscript revision, read, and approved the submitted manuscript.

## Conflict of Interest

The authors declare that the research was conducted in the absence of any commercial or financial relationships that could be construed as a potential conflict of interest.
